# Comparison of small-area deprivation measures as predictors of chronic disease burden in a low-income population

**DOI:** 10.1186/s12939-016-0378-9

**Published:** 2016-06-10

**Authors:** Ana Lòpez-De Fede, John E. Stewart, James W. Hardin, Kathy Mayfield-Smith

**Affiliations:** Institute for Families in Society, University of South Carolina, 1600 Hampton Street, Suite 507, Columbia, 29208 SC USA; Department of Epidemiology and Biostatistics, Arnold School of Public Health, University of South Carolina, 915 Greene Street, Room 445, Columbia, SC USA

**Keywords:** Small-area deprivation, Chronic disease, Low-income population

## Abstract

**Background:**

Measures of small-area deprivation may be valuable in geographically targeting limited resources to prevent, diagnose, and effectively manage chronic conditions in vulnerable populations. We developed a census-based small-area socioeconomic deprivation index specifically to predict chronic disease burden among publically insured Medicaid recipients in South Carolina, a relatively poor state in the southern United States. We compared the predictive ability of the new index with that of four other small-area deprivation indicators.

**Methods:**

To derive the ZIP Code Tabulation Area-Level Palmetto Small-Area Deprivation Index (Palmetto SADI), we evaluated ten census variables across five socioeconomic deprivation domains, identifying the combination of census indicators most highly correlated with a set of five chronic disease conditions among South Carolina Medicaid enrollees. In separate validation studies, we used both logistic and spatial regression methods to assess the ability of Palmetto SADI to predict chronic disease burden among state Medicaid recipients relative to four alternative small-area socioeconomic deprivation measures: the Townsend index of material deprivation; a single-variable poverty indicator; and two small-area designations of health care resource deprivation, Primary Care Health Professional Shortage Area and Medically Underserved Area/Medically Underserved Population.

**Results:**

Palmetto SADI was the best predictor of chronic disease burden (presence of at least one condition and presence of two or more conditions) among state Medicaid recipients compared to all alternative deprivation measures tested.

**Conclusions:**

A low-cost, regionally optimized socioeconomic deprivation index, Palmetto SADI can be used to identify areas in South Carolina at high risk for chronic disease burden among Medicaid recipients and other low-income Medicaid-eligible populations for targeted prevention, screening, diagnosis, disease self-management, and care coordination activities.

## Background

In the United States persons with chronic conditions are overrepresented in Medicaid [1], a publically funded social health insurance program for persons with low incomes and limited resources [2]. Policy and programming efforts to control spending and improve health outcomes among Medicaid enrollees must address the health care requirements of high-need, high-cost recipients with chronic diseases. Low-cost small-area assessment tools based on existing data may be especially valuable in geographically targeting limited resources to prevent, diagnose, and effectively manage chronic conditions in high-risk Medicaid populations.

Increasingly, small-area measures of social and material deprivation [3] are used to discern geographic patterns of morbidity [4, 5] and mortality [6, 7]. The utilization of these measures in health research is theoretically grounded in internationally recognized social determinants of health literature, which consistently identifies worse health outcomes in socioeconomically disadvantaged communities [8]. One such measure, the Townsend deprivation index, has been used widely in population health studies. Developed in the United Kingdom, this small-area deprivation measure consists of four census-based component indicators reflecting local levels of unemployment, home ownership, household crowding, and vehicle availability [9]. The Townsend deprivation index has been used to evaluate associations between community deprivation and such diverse health outcomes as bacteremic pneumonia [10], tuberculosis [5, 11], sexually transmitted infections [5], infant mortality [7], and motor vehicle deaths [12]. Similarly, a single-variable poverty index (proportion of the population living below a designated poverty level) has been used extensively in studies exploring associations between community deprivation and poor health. Poverty rates have been employed, for instance, as neighborhood-level predictors of low birth weight [13], AIDS [14], tuberculosis [5, 11], pneumonia [10], stroke mortality [15], and all-cause mortality [16]. Several investigators have noted worse health outcomes in areas lacking sufficient numbers of health care providers [17–19]. Two US Health Resources and Services Administration (HRSA) small-area health care resource deprivation designations—Primary Care Health Professional Shortage Area (PC-HPSA) and Medically Underserved Area/Medically Underserved Population (MUA/MUP) [20]—thus also might prove useful in identifying US communities at risk for poor health.

Although the Townsend deprivation index, single-variable poverty index, and health care resource deprivation designations are used widely in health planning and evaluation, these measures may not be optimally suited for purposes of community health need assessment in all geographic regions or across diverse population groups. Indeed, a marked trend exists in the development of region/population-specific small-area deprivation indexes for health research. Since 2000, for example, deprivation measures have been constructed and applied in health studies in Quebec, Canada [21]; Verona, Northern Italy [22]; France [23, 24]; Australia [25]; Puerto Rico [26]; Switzerland [27]; Denmark [28]; Sweden [29]; Nova Scotia, Canada [30]; and Quito City, Ecuador [31]. Six of these measures were introduced in just four years between 2012 and 2015 [26–31].

**Fig. 1:**
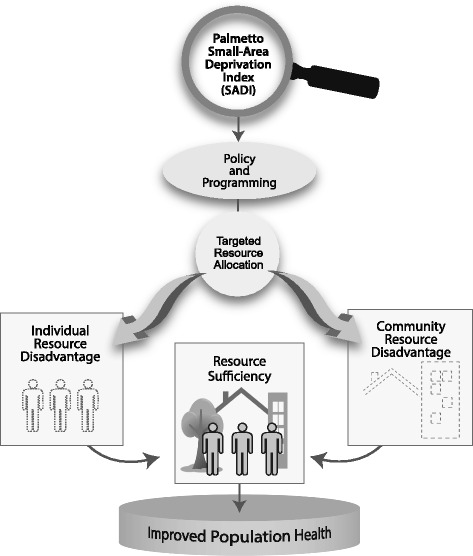
Palmetto Small-Area Deprivation Index (SADI) conceptual framework. Based on Lu Ann Aday’s “Framework for studying vulnerable populations.” (Aday LA. At risk in America: the health and health care needs of vulnerable populations in the United States. San Francisco, CA: Jossey-Bass; 2001)

To our knowledge, no socioeconomic deprivation measure has been developed specifically for assessment of a Medicaid population in the United States. To facilitate health policy and programming, we developed a census-based small-area socioeconomic deprivation index optimized to predict chronic disease burden among Medicaid recipients in South Carolina, a largely impoverished Southern state where more than one in five residents are enrolled in the Medicaid system [32]. Based on the conceptual framework of Aday [33], this index measures community-level resource deprivation that puts low-income Medicaid enrollees and other vulnerable individuals at increased risk for poor health. Information derived from the index can help state agencies, health care providers, non-profit organizations and community groups better target limited social, economic, and health care resources to improve population health (Fig. 1). In this paper we describe the construction of the new index, the Palmetto Small-Area Deprivation Index (Palmetto SADI); compare its ability to predict Medicaid population chronic disease burden with that of four alternative small-area deprivation measures; and identify its potential to strengthen chronic disease prevention, screening, diagnosis, self-management, and care coordination activities for at-risk populations. Our study illustrates the development of a region/population-specific, census-based small-area deprivation measure and shows that such an optimized index can outperform other widely employed deprivation indicators in predicting region/population-specific health outcomes.

## Methods

### Deprivation index construction

The US Census Bureau provides detailed population and housing data at multiple geographic levels. US census and survey data products are updated regularly and are available online at no cost, making them especially valuable to state and local health planners with limited financial resources. We sought to create a census-based index of socioeconomic deprivation to predict chronic disease burden among South Carolina Medicaid enrollees at the ZIP Code Tabulation Area (ZCTA) level. Census-defined ZCTAs are comprised of whole census blocks and spatially approximate USPS five-digit ZIP Code mail delivery areas [34]. These small-area units have served as proxies for residential neighborhoods in previous health studies [11, 35–37]. ZCTAs are appropriate units of analysis when, as in our case, residential address limitations (missing, incomplete or invalid street address data) prevent the geolocation and evaluation of spatial data at finer scales (e.g., across census tracts or census block groups). There are 424 ZCTAs in South Carolina with an average population of about 10,800 persons [38].

Based on a literature review, we evaluated a range of Census 2000 population and housing indicators [39] for inclusion in the deprivation index (Table 1). We assessed two variables in each of five distinct socioeconomic domains: education (percentage of persons 25 years and older without a high school diploma, percentage of persons 16 to 19 years not enrolled in school and not a high school graduate); income (percentage of noninstitutionalized population below the federal poverty level, percentage of households with income less than $15,000); employment (percentage of persons 16 and older unemployed, percentage of persons 16 to 64 working part-time); social fragmentation (percentage of persons 15 and older unmarried or separated, percentage of families with own children under 18 years headed by a single female); and material deprivation (percentage of housing units that are renter-occupied, percentage of housing units with no vehicle available). These five domains have been identified previously as relevant dimensions of small-area socioeconomic deprivation and have been consistently operationalized by others using the same or similar census measures [4, 9, 40, 41].

**Table 1:** Index construction: census socioeconomic and chronic condition indicators (ZCTA level)

Census socioeconomic indicators^a^	Minimum	Maximum	Mean	Standard deviation
% Persons 25 and Older Without a HS Diploma	3.1	57.3	28.4	10.1
% Persons 16–19 Not Enrolled and Not a HS Graduate	0.0	100.0	12.4	10.2
% Persons Below Poverty Level	0.0	41.1	15.9	7.3
% Households With Income < $15,000	0.0	45.2	21.8	8.5
% Persons 16 and Older Unemployed	0.0	16.4	6.3	3.0
% Persons 16–64 Working Part-Time	0.0	47.6	17.5	4.9
% Persons 15 and Older Unmarried or Separated	12.2	80.2	45.6	8.5
% Single Female-Headed Family Households	0.0	100.0	25.2	11.3
% Renter-Occupied Households	0.0	100.0	22.8	12.9
% Households With No Vehicle	0.0	41.4	9.9	5.8
Prevalence Rates per 1,000 Medicaid Enrollees^b^	Minimum	Maximum	Mean	Standard Deviation
Cardiovascular Disease	0.0	216.9	46.2	19.6
Diabetes	0.0	169.3	65.1	25.4
End-Stage Renal Disease	0.0	100.5	12.6	8.6
Hypertension	31.7	333.3	113.5	42.7
Obesity	0.0	73.2	19.5	8.5

The table presents ZCTA-level summary statistics for census-based socioeconomic indicators and Medicaid chronic condition prevalence rates evaluated in the construction of a new small-area deprivation index. Values represent all South Carolina ZCTAs for which complete data were available (*N* = 384)

Sources: ^a^U.S. Census 2000 SF3; ^b^SC Medicaid Management Information System, FY2010

We evaluated chronic disease burden among South Carolina Medicaid recipients across five adverse chronic health conditions: cardiovascular disease (CVD); diabetes; end-stage renal disease (ESRD); hypertension; and obesity. These diagnostic categories are among the most common and costliest chronic conditions affecting South Carolina Medicaid enrollees. Chronic disease status for the state’s approximately 1 million Medicaid recipients was determined using primary and secondary diagnosis codes contained in South Carolina Medicaid administrative data sets from fiscal year 2010 (July 2009 to June 2010) [42]. ZCTA-level prevalence rates per 1,000 Medicaid enrollees were calculated for each chronic condition (Table 1).

In developing the new socioeconomic deprivation index, we sought to minimize the total number of census-based predictor variables while maximizing correlation with ZCTA-level Medicaid chronic disease rates. We scaled each predictor (*X*_*i*_) using Fisher’s Z-transformation to create a set of Z-score variables (*Z*_*i*_) defined for *n*_*i*_ observations *j* = 1,…,*n*_*i*_ based on the associated original variable

$$ {Z}_{ij}=\frac{X_{ij}-{\overline{\overline{X}}}_i}{\sqrt{{\displaystyle {\sum}_{k=1}^{n_i}}{\left({X}_{ik}-{\overline{\overline{X}}}_i\right)}^2/\left({n}_i-1\right)}} $$

where ${\overline{\overline{X}}}_i$ is the sample mean of the *i*th predictor. This transformation ensures that each of the Z-score variables is standardized to have mean 0 and variance 1. We then calculated the mean correlation of each transformed variable across the set of five chronic condition prevalence rates. The single predictor with the highest mean correlation was the first component $\left\{{X}_{i_1}\right\}$ included in the index. Thus, the best single predictor index, ${\mathrm{S}}_1\left\{{X}_{i_1}\right\}$, was defined as

$$ {S}_1\left\{{X}_{i_1}\right\}\ \ge\ {S}_1\left\{{X}_j\right\}\ \mathrm{f}\mathrm{o}\mathrm{r}\ j=1,\dots,\ 10 $$

Additional variables were included only if the new measure represented a domain not yet in the index

$$ {S}_2\left\{{X}_{i_1},{X}_{i_2}\right\}\ge {S}_2\left\{{X}_{i_1},{X}_j\right\}\kern0.75em \mathrm{f}\mathrm{o}\mathrm{r}\ j=1,\dots, 10\kern0.5em \mathrm{and}\kern0.5em \mathrm{Domain}\left({X}_{i_2}\right)\ne \mathrm{Domain}\left({X}_{i_1}\right) $$

Further, ${S}_{k+1}\left\{{X}_{i_1},\dots, {X}_{i_{k+1}}\right\}$ was preferred over ${S}_k\left\{{X}_{i_1},\dots, {X}_{i_k}\right\}$ only if including the new variable ${X}_{i_{k+1}}$ increased the resulting index’s mean correlation with the set of five chronic conditions (*Cond*_*i*_)

$$ \frac{1}{5}{\displaystyle \sum_{i=1}^5}\mathrm{Corr}\left({S}_{k+1}\left\{{X}_{i_1},\dots, {X}_{i_{k+1}}\right\}, Con{d}_i\right) > \frac{1}{5}{\displaystyle \sum_{i=1}^5}\mathrm{Corr}\left({S}_k\left\{{X}_{i_1},\dots, {X}_{i_k}\right\}, Con{d}_i\right) $$

In constructing the index we considered only ZCTAs with complete attribute data across all ten census variables evaluated and for which Medicaid chronic disease prevalence rates could be calculated (*N* = 392).

Thus developed, the final deprivation index, Palmetto SADI, consisted of three component variables: percentage of persons 25 years and older without a high school diploma, percentage of noninstitutionalized persons below the federal poverty level, and percentage of housing units with no vehicle available. In a factor analysis of all predictors, the three variables comprising the new index loaded on a single factor. The component variable loading scores were nearly identical; we thus considered each of the components to be of equal weight in its contribution to the overall index score. ZCTA-level index scores were derived by summing ZCTA-specific Z-scores for each component variable. Additive Z-score methods have been employed in the construction of other socioeconomic deprivation measures [24], including the widely known Townsend index. Had the factor analysis identified multiple factors or had the components loaded differentially, component variable weighting might have been indicated. That there was a single factor with similar loadings is consistent with the summative Z-score approach used.

A number of alternative methods have been used to construct small-area socioeconomic deprivation measures [24, 31]. We investigated the selection of deprivation index component variables using boosted regression methods based on regression forests. Boosted regression, or boosting, is a statistical learning algorithm that averages the results of large numbers of decision trees (forests) to derive predicted values. This data mining algorithm has proven valuable in wide-ranging health studies, including investigations of dengue transmission [43], gene expression [44], and complex epidemiologic interaction effects [45]. Using boosting methods, we estimated the relative influence of each of the ten socioeconomic covariates (two variables in five socioeconomic domains) in predictive models of each of the five chronic disease outcomes identified previously. Allowing for 20,000 possible models, we selected the three most influential socioeconomic covariates across all five chronic disease outcomes. This method yielded a composite index identical to Palmetto SADI in its representation of socioeconomic domains (education, income, and material deprivation), with nearly identical component variables (percentage of persons 25 years and older without a high school diploma, percentage of noninstitutionalized population below the federal poverty level, and percentage of housing units that are renter-occupied). The boosted regression-based model, however, did not perform as well as Palmetto SADI in validation studies and thus was rejected as a candidate deprivation measure.

### Comparison of small-area deprivation measures

To validate the new index, we tested the ability of Palmetto SADI to predict chronic disease burden among Medicaid recipients, using more recent data sets. Assessments of predictive validity have been used widely to establish the quality of deprivation indexes [46, 47]. The predictive capacity of Palmetto SADI was evaluated relative to four alternative measures: two socioeconomic deprivation indicators (the Townsend index and a single-variable poverty measure) and two small-area HRSA designations of health care resource deprivation (PC-HPSA and MUA/MUP). ZCTA-level Palmetto SADI, Townsend index, and poverty scores were derived using data from the US Census Bureau, American Community Survey (ACS) 2007–2011 5-Year Estimates [38]. PC-HPSA and MUA/MUP data representing the year 2012 were obtained from the US Department of Health and Human Services, Health Resources and Services Administration [20]. ZCTAs with population centroids located within federally designated PC-HPSAs and/or MUAs/MUPs were classified accordingly. South Carolina Medicaid administrative data from fiscal year 2012 (July 2011 to June 2012) were used to identify chronic disease status for state Medicaid enrollees [48].

We first tested the capacity of Palmetto SADI to predict chronic disease burden among a random sample of Medicaid enrollees as measured across five selected conditions (CVD, diabetes, ESRD, hypertension, and obesity). Two chronic disease burden indicators—one reflecting the presence of at least one chronic condition and the other representing the presence of two or more conditions—were created for a random sample of 5,000 Medicaid recipients geocoded at the ZCTA level using recipient residential address data. Utilizing this sample, we performed logistic regression analyses to evaluate the ability of Palmetto SADI and four alternative measures of small-area deprivation to predict chronic disease burden among Medicaid enrollees based on their ZCTA of residence.

$$ \mathrm{Model}\ 1:\kern0.5em {\mathrm{logit}}^{-1}\left({y}_i\right) = {\beta}_0^{\left[1\right]} + {\beta}_1^{\left[1\right]}\mathrm{Palmetto}\ {\mathrm{SADI}}_i $$

$$ \mathrm{Model}\ 2:\kern0.5em {\mathrm{logit}}^{-1}\left({y}_i\right) = {\beta}_0^{\left[2\right]} + {\beta}_1^{\left[2\right]}{\mathrm{Townsend}}_i $$

$$ \mathrm{Model}\ 3:\kern0.5em {\mathrm{logit}}^{-1}\left({y}_i\right) = {\beta}_0^{\left[3\right]} + {\beta}_1^{\left[3\right]}{\mathrm{Poverty}}_i $$

$$ \mathrm{Model}\ 4:\kern0.5em {\mathrm{logit}}^{-1}\left({y}_i\right) = {\beta}_0^{\left[4\right]} + {\beta}_1^{\left[4\right]}\mathrm{P}\mathrm{C}\hbox{-} {\mathrm{HPSA}}_i $$

$$ \mathrm{Model}\ 5:\kern0.5em {\mathrm{logit}}^{-1}\left({y}_i\right) = {\beta}_0^{\left[5\right]} + {\beta}_1^{\left[5\right]}\mathrm{M}\mathrm{U}\mathrm{A}/{\mathrm{MUP}}_i $$

In these analyses Palmetto SADI, Townsend, and poverty were evaluated as continuous measures; PC-HPSA and MUA/MUP were modeled as binomial variables. We evaluated the performance of all models using the area under the Receiver Operating Characteristic curve (AUC). This statistic summarizes a model’s discrimination, i.e., ability to correctly classify individuals’ chronic disease status. AUC values close to 1 show near perfect discrimination. The model fit was evaluated using the corrected Akaike information criterion measure (AIC). This is a measure of the model’s deviance or difference from a saturated (perfectly predicting) model. Lower values of AIC indicate a preferable model. Bootstrapping was used to estimate standard errors of the AUC and AIC values which allowed assessment of significant differences across models; by this approach, we generated 199 random samples (with replacement) from the original data and re-estimated each of the five models. Approximate standard errors were given by the standard deviation of results from the bootstrap samples. For example, the standard error of the observed area under the curve *AUCO*_*o*_ is

$$ SE\left( AU{C}_o\right) = \sqrt{\frac{1}{198}{\displaystyle {\sum}_{i=1}^{199}}{\left( AU{C}_i- AU{C}_o\right)}^2} $$

where *AUC*_*i*_ is the area under the curve of the model estimated from the *i*th bootstrap sample.

Next, we derived ZCTA-level total Medicaid population and chronic disease counts for each of the five chronic conditions represented in logistic regression analyses, based on georeferenced data for the entire Medicaid population (*N* = 1,024,034). We further derived two ZCTA-level chronic disease burden counts (presence of at least one chronic condition and presence of two or more conditions). We calculated odds ratios to assess associations between high socioeconomic deprivation as measured by Palmetto SADI, the Townsend index, and the poverty measure (top versus bottom quartile of each continuous deprivation measure distribution) and each of the seven chronic condition indicators (five single conditions, presence of any condition, presence of two or more conditions). Similarly, we calculated odds ratios to evaluate associations between two binomial measures of health care provider resource deprivation (PC-HPSA, MUA/MUP) and each of the seven chronic condition measures.

We performed Ordinary Least Squares (OLS) and spatial regression analyses to further evaluate small-area deprivation measure associations with chronic disease burden at the ZCTA level, again based on georeferenced data for the entire Medicaid population. Chronic disease prevalence rates were calculated for five conditions (asthma, CVD, diabetes, ESRD, and hypertension). Two chronic disease burden prevalence rates (presence of at least one chronic condition and presence of two or more conditions) also were calculated. As in previous logistic regression analyses, Palmetto SADI and four alternative measures of small-area deprivation were modeled. Preliminary OLS regression analyses with spatial diagnostics (Moran’s I) indicated statistically significant spatial autocorrelation in all models tested. Spatial regression models (spatial lag or spatial error models as indicated by Lagrange Multiplier test statistics) were employed to account for the spatial autocorrelation of modeled variables. Spatial regression results are reported. AIC and Schwarz Bayesian information criterion (BIC) values from spatial regressions were used to evaluate goodness of fit for each small-area deprivation model, with lower values indicating preferable models. To ensure greater prevalence rate stability and protect recipient confidentiality in mapped results, all ZCTA-level index validation analyses were restricted to ZCTAs with at least 30 Medicaid enrollees (*N* = 372). The operationalization of small-area deprivation measures for this set of ZCTAs is summarized in Table 2. Logistic regression modeling and bootstrapping procedures were performed using Stata software Version 12 [49]. OLS and spatial regressions were conducted using GeoDa version 1.6 [50]. All geoprocessing was performed using ESRI ArcGIS 10.2 [51].

**Table 2:** Small-area deprivation measure operationalization (ZCTA Level)

Measure	Type	Range (Number of ZCTAs)			
		Bottom quartile	Second quartile	Third quartile	Top quartile
Palmetto SADI^a^	Additive Z-Score Composite/Continuous	−5.10 to −1.77 (93)	−1.76 to −0.10 (93)	−0.09 to 1.55 (93)	1.56 to 7.76 (93)
Townsend^a^	Additive Z-Score Composite/Continuous	−4.86 to −1.78 (93)	−1.77 to −0.21 (93)	−0.20 to 1.36 (93)	1.37 to 12.35 (93)
Poverty^a^	Single Variable/Continuous	0.0 to 12.5 (92)	12.6 to 17.9 (94)	18.0 to 24.4 (93)	24.5 to 64.0 (93)
		Class (Number of ZCTAs)			
PC-HPSA^b^	Single Variable/Binomial	Not a Designated Area (76)	Designated Area (296)		
MUA/MUP^b^	Single Variable/Binomial	Not a Designated Area (118)	Designated Area (254)		

Tabled values represent South Carolina ZCTAs with at least 30 Medicaid enrollees (*N* = 372)

Sources: ^a^U.S. Census, ACS 2007–2011 5-Year Estimates; ^b^HRSA, 2012

## Results

**Fig. 2:**
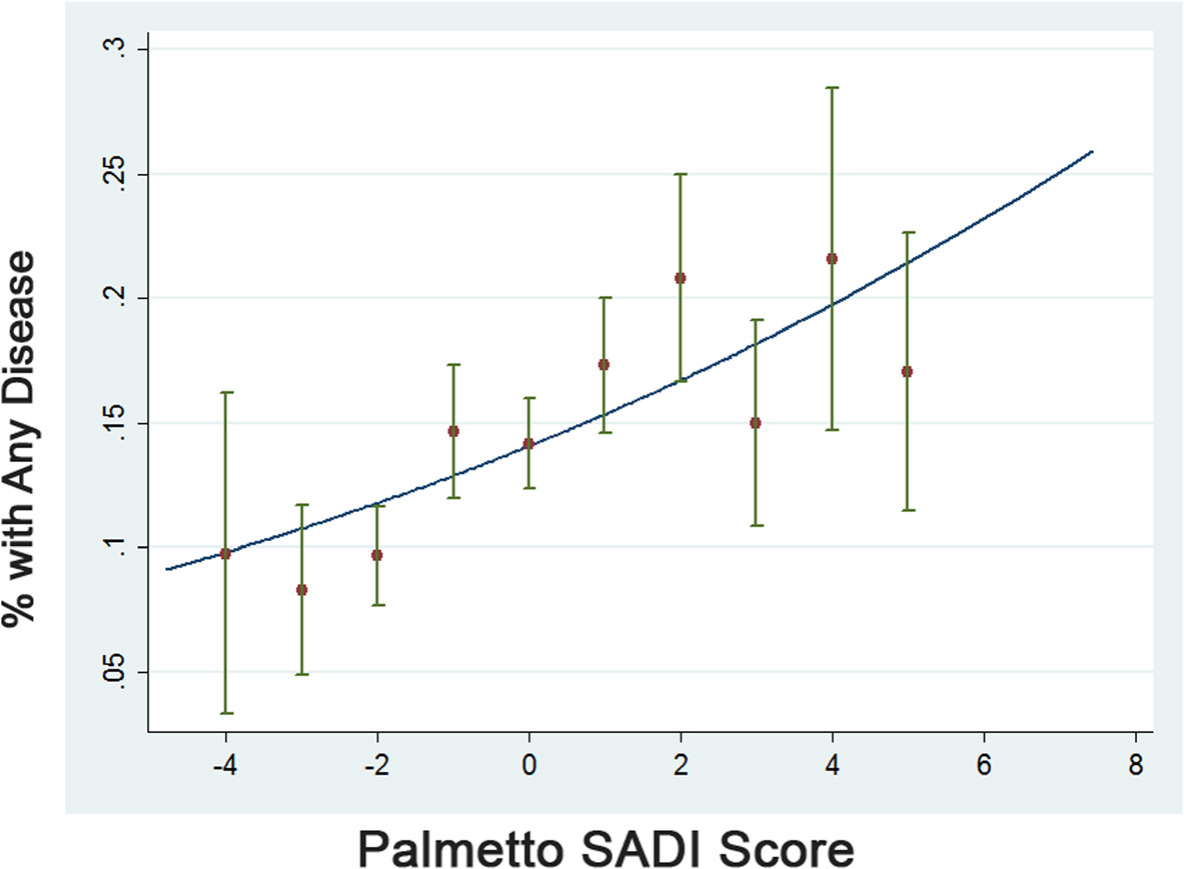
Observed versus predicted probability of chronic disease burden by Palmetto SADI score

Approximately 15 % of all South Carolina Medicaid recipients had at least one of the five chronic conditions considered in the construction of the deprivation index; nearly 6 % had two or more conditions. Figure 2 illustrates a clear association between observed rates of chronic disease burden (as indicated by the presence of at least one select chronic condition) among a random sample of Medicaid enrollees and the predicted probability of chronic disease burden based on ZCTA-level socioeconomic deprivation as measured by Palmetto SADI (observed rates are depicted as dots with associated 95 % confidence intervals; a curved line represents the predicted probability). In logistic regression analyses based on a random sample of 5,000 Medicaid recipients, Palmetto SADI was a better predictor of chronic disease burden (presence of at least one chronic condition and presence of two or more conditions) than the Townsend index, poverty measure, PC-HPSA designation, and MUA/MUP designation. The Palmetto SADI model had a significantly higher AUC (*P* < 0.001) and a significantly lower AIC (*P* < 0.001) compared to all four alternative models (Table 3). In separately performed age category analyses, Palmetto SADI was the best predictor of chronic disease burden (at least one chronic condition, two or more chronic conditions) in adult Medicaid recipients and the overall best predictor of chronic disease burden among child Medicaid beneficiaries as measured across three chronic conditions affecting children—asthma, diabetes, and obesity (there was no statistical difference between the two best predictors of any chronic condition in children, Palmetto SADI and the Townsend index; nor was there any statistical difference between the two best predictors of comorbidity, Palmetto SADI and the poverty measure).

**Table 3:** Logistic regression AUC and AIC values: Palmetto SADI versus four alternative small-area deprivation measures

Model	At least one chronic condition				Two or more chronic conditions			
	AUC	p	AIC	p	AUC	p	AIC	p
Palmetto SADI	0.5741		4080.450		0.5716		2192.305	
Townsend	0.5472	0.0000	4101.994	0.0000	0.5416	0.0000	2200.699	0.0000
Poverty	0.5563	0.0000	4095.502	0.0000	0.5528	0.0000	2198.326	0.0000
Primary Care HPSA	0.5440	0.0000	4095.045	0.0000	0.5419	0.0000	2197.537	0.0000
MUA/MUP	0.5389	0.0000	4101.473	0.0000	0.5299	0.0000	2202.166	0.0000

The table shows omnibus statistics for single predictor logistic regression models of two chronic disease burden outcome indicators representing a random sample of 5,000 South Carolina Medicaid recipients (FY2012). Area under the Receiver Operating Characteristic curve (AUC) values close to 1 show near perfect discrimination. Corrected Akaike information criterion measure (AIC) values indicate the model’s deviance from a perfectly predicting model. Lower values of AIC indicate a preferable model. Tabled *p* values reflect the probability that AUC and AIC values associated with each of the four alternative deprivation models do not differ statistically from the Palmetto SADI model

Unadjusted odds ratios indicated significantly higher levels of chronic disease in high- versus low-deprivation ZCTAs, regardless of the deprivation indicator used. For all chronic conditions but obesity, the observed odds ratios were highest when Palmetto SADI was used to identify high-deprivation areas. Likewise, odds ratios for both chronic disease burden indicators (at least one chronic condition, two or more chronic conditions) were highest when Palmetto SADI was used to identify high socioeconomic deprivation (Table 4).

**Table 4:** ZCTA-level association of socioeconomic deprivation/health care resource deprivation measures with selected chronic condition prevalence rates

Chronic condition	Palmetto SADI High Deprivation^a^ Odds ratio (95 % CI)	Townsend High Deprivation^a^ Odds ratio (95 % CI)	High poverty^b^ Odds ratio (95 % CI)	Primary care HPSA^c^ Odds ratio (95 % CI)	MUA/MUP^d^ Odds ratio (95 % CI)
CVD	1.69 (1.64, 1.74)	1.37 (1.33, 1.42)	1.59 (1.54, 1.64)	1.31 (1.28, 1.35)	1.21 (1.18, 1.23)
Diabetes	1.91 (1.86, 1.96)	1.52 (1.48, 1.56)	1.80 (1.76, 1.85)	1.39 (1.36, 1.42)	1.37 (1.34, 1.39)
ESRD	2.20 (2.08, 2.33)	1.76 (1.65, 1.87)	2.05 (1.93, 2.17)	1.23 (1.18, 1.28)	1.29 (1.24, 1.34)
Hypertension	2.11 (2.07, 2.15)	1.71 (1.67, 1.74)	2.01 (1.97, 2.05)	1.39 (1.37, 1.42)	1.40 (1.38, 1.42)
Obesity	1.26 (1.21, 1.31)	1.28 (1.22, 1.33)	1.32 (1.26, 1.37)	1.05 (1.02, 1.08)	1.05 (1.02, 1.08)
Any Condition	1.82 (1.79, 1.85)	1.52 (1.49, 1.54)	1.74 (1.71, 1.76)	1.33 (1.31, 1.34)	1.29 (1.28, 1.31)
Two or More Conditions	2.17 (2.12, 2.23)	1.71 (1.66, 1.76)	2.08 (2.02, 2.13)	1.40 (1.37, 1.43)	1.40 (1.38, 1.43)

^a^High deprivation is defined as the highest quartile of the ZCTA-level deprivation index score distribution; referent = lowest quartile

^b^High poverty is defined as the highest quartile of the ZCTA-level poverty prevalence distribution; referent = lowest quartile

^c^HPSA designated versus non-designated ZCTAs

^d^MUA/MUP designated versus non-designated ZCTAs

Consistent with logistic regression results, spatial regression analyses identified Palmetto SADI as the best small-area deprivation predictor of chronic disease burden (at least one condition, two or more conditions) among all Medicaid recipients at the ZCTA level. Compared to the four alternative deprivation models tested, the Palmetto SADI model yielded lower AIC and BIC values, thus indicating the preferability of the derived index (Table 5). Separate age category analyses showed Palmetto SADI was the best predictor of any chronic disease and multiple chronic conditions among adult Medicaid recipients. For child Medicaid beneficiaries, there was no substantial difference between the two best small-area deprivation measures, Palmetto SADI and the Townsend index, as predictors of childhood chronic disease burden. The lack of discrimination between these two deprivation indicators likely reflects the low prevalence of chronic disease measured among child enrollees.

**Table 5:** ZCTA-level spatial regression model statistical criteria: Palmetto SADI versus four alternative small-area deprivation measures

Model	At least one chronic condition			Two or more chronic conditions		
	R-squared	AIC	BIC	R-squared	AIC	BIC
Palmetto SADI	0.6224	3608.91	3620.67	0.6117	3203.39	3215.15
Townsend	0.5843	3662.31	3674.06	0.5754	3251.79	3263.55
Poverty	0.5727	3662.86	3674.62	0.5864	3232.06	3243.81
Primary Care HPSA	0.5454	3693.40	3705.16	0.5614	3262.62	3274.38
MUA/MUP	0.5457	3692.42	3704.17	0.5635	3257.63	3269.38

The table shows omnibus statistics for ZCTA-level single predictor spatial regression models of two chronic disease burden indicators derived for all FY2012 South Carolina Medicaid recipients (*N* = 1,024,034). Akaike information criterion measure (AIC) and Schwarz Bayesian information criterion measure (BIC) values indicate the model’s deviance from a perfectly predicting model. Lower values of AIC and BIC indicate a preferable model

**Fig. 3:**
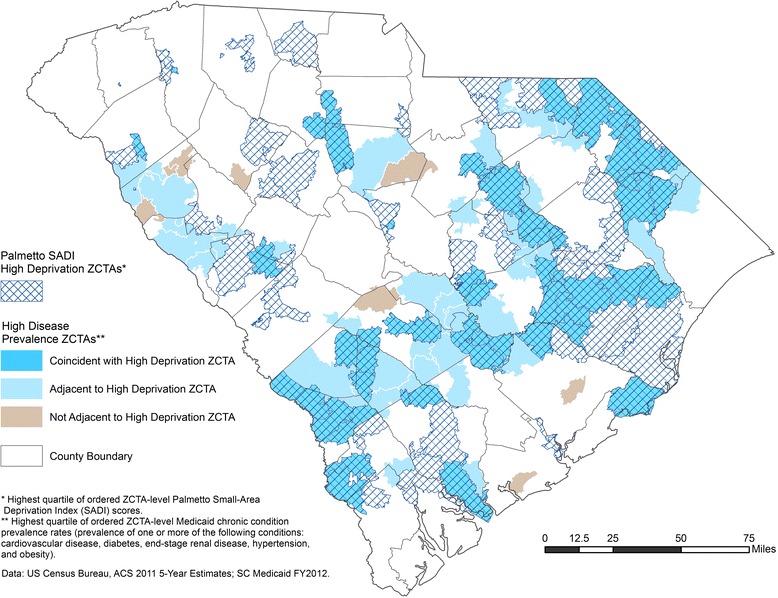
Palmetto SADI high-deprivation and high disease prevalence ZIP Code Tabulation Areas in South Carolina

Figure 3 shows the geographic distribution of Palmetto SADI high deprivation ZCTAs (top quartile of ordered ZCTA-level Palmetto SADI scores) and high disease prevalence ZCTAs (top quartile of ordered ZCTA-level chronic disease burden rates, prevalence of at least one chronic condition) in South Carolina. Substantial spatial coincidence of high deprivation and high disease prevalence areas exists. If not geographically coincident, high disease prevalence areas typically adjoin Palmetto SADI high-deprivation areas.

## Discussion

We found significantly higher levels of chronic disease in high- versus low-deprivation ZCTAs, regardless of the deprivation measure used, a result that is consistent with a growing international body of literature indicating higher rates of wide-ranging adverse health outcomes in resource-poor communities [4, 5, 8, 11, 29, 30, 52]. Notably, the highest odds ratios for chronic disease burden were associated with the Palmetto SADI operationalization of small-area socioeconomic deprivation. In both logistic and spatial regression analyses, the Palmetto SADI model was the best overall predictor of chronic disease burden (any condition and two or more conditions) among South Carolina Medicaid enrollees, compared to four alternative small-area deprivation models. Our results indicate the widely used Townsend index and single-variable poverty index are not always the best small-area deprivation measures by which to identify at-risk populations for targeted health interventions. Similarly, we found HRSA PC-HPSAs and MUAs/MUPs less predictive of chronic disease burden than Palmetto SADI, a finding in line with calls in the United States to revise HPSA and MUA designation criteria to better reflect population health care need, in addition to provider supply and demand [53]. The ability of Palmetto SADI to accurately identify areas of high chronic disease burden is of value to policy and decision makers responsible for the geographic allocation of limited health care resources. Resource allocation efficiency, however, also requires that the inaccurate identification of high burden areas by the index be minimized (i.e., the measure’s false positive rate should be low). Utilizing a model-specific cutoff value to ensure equality of means, we calculated the false positive rates of Palmetto SADI and the four alternative deprivation measures in identifying areas of high chronic disease burden (presence of any condition). Of the measures tested, Palmetto SADI had the lowest false positive rate (15.8 %); the Townsend index had the second lowest rate (17.6 %).

Although small-area deprivation measures have proven useful in geospatial assessments of population health and health inequality, such measures are subject to criticism, particularly in terms of variable selection and index construction [6]. We based our initial selection of ten candidate variables on a review of relevant literature. All of the variables we considered as index components represent widely recognized socioeconomic deprivation domains [4, 9, 40, 41]. Our decision to weight each of the component variables equally in an additive Z-score index was based on the results of a factor analysis in which all three variables loaded on a single factor with nearly identical loading scores. Our exploration of an alternative construction method failed to yield a superior index. Ultimately, the construction of a deprivation index must be consistent with clearly defined planning and policy goals [54]. With this guideline in mind, we developed Palmetto SADI specifically to identify areas of high chronic disease burden among South Carolina Medicaid recipients. The high predictive validity [47] of the derived index established in logistic and spatial regression analyses demonstrates the measure’s quality and potential to inform Medicaid chronic care policy and planning at state and local levels.

Beyond the recognition of conceptual and methodological challenges associated with the construction of any socioeconomic deprivation measure, several limitations specific to the development, validation, and application of Palmetto SADI should be identified. First, chronic disease status was determined using diagnostic codes in Medicaid administrative data sets. Administrative data are widely used in health studies and the validity of such data sets has been established [55]. More accurate information about individual recipient health status, however, might be derived from patient clinical records. Second, behavioral health disorders were not considered in the development of the index. Further research is needed to evaluate the ability of Palmetto SADI to predict such chronic behavioral conditions as ADHD and depression. Third, index validation analyses only included ZCTAs with 30 or more Medicaid enrollees. The ability of the new index relative to other deprivation measures to predict chronic disease burden in very small Medicaid population areas thus remains uncertain. Fourth, the ZCTA-level Palmetto SADI does not permit evaluation of chronic disease burden at finer geographic scales. Residential address quality issues (missing, incomplete, or invalid street address information) prevented us from georeferencing Medicaid recipients at census tract or census block group levels. More than 98% of recipients, however, could be geocoded at the ZCTA level. Caution should be exercised in the use of ZCTAs in health systems research, particularly because postal ZIP Codes and census ZCTAs do not always correspond, either in nominal or spatial terms [56]. In this study we minimized potential ZCTA-level geocoding errors by using street address data whenever available and by using both ZIP and ZIP-plus-4 centroid coordinate data when street address information was missing or incomplete. Lastly, the new index was constructed specifically to predict chronic disease burden among South Carolina Medicaid enrollees. Further research is needed to evaluate the utility of the index for this or similar analytic purposes in neighboring Southern states and other geographic regions.

As indicated by specific policy or programming requirements, the methodology described might be used to construct census-based socioeconomic deprivation measures for both smaller (e.g., census tract, census block group) and larger (e.g., hospital referral region, county) areas. “Tailored” deprivation indexes [22] also might be created to predict chronic disease burden or other health conditions among different subpopulations (e.g., children, older adults, or women). As this study illustrates, user-derived, census-based small-area deprivation measures can outperform such widely employed deprivation indicators as the Townsend index and single-variable poverty measure in predicting region/population-specific health outcomes.

The development of Palmetto SADI is consistent with calls for better measures of social and health deprivation that permit the identification and reduction of health disparities across time and space [57] and that inform decisions regarding the geographic allocation of health resources [53]. The derivation of the new index parallels the construction of other recent region/population-specific small-area deprivation measures for health research [26–31]. Palmetto SADI is the first socioeconomic deprivation index developed specifically to inform policy and programming for a US Medicaid population. The new index can be introduced to public health and health care stakeholders in South Carolina as regionally relevant and straightforward in interpretation, thereby encouraging support for—and actual utilization of—the information tool. Palmetto SADI can be used to identify areas at high risk for chronic disease burden among Medicaid recipients and other Medicaid-eligible low-income populations for targeted prevention, screening, diagnosis, disease self-management, and care coordination activities. Our spatial visualization results suggest that in many instances such intervention efforts could appropriately be extended into areas immediately surrounding (adjacent to) high-deprivation neighborhoods. Geographically targeted interventions aimed at early diagnosis, appropriate disease management, and effective care coordination all can improve chronic disease outcomes and may yield health care cost savings by reducing patient emergency room visits, hospitalizations, hospital readmissions, and unnecessary prescription drug use [58, 59]. Coordinated and continuous chronic disease management also may slow disease progression, allowing patients to maintain functional status [55] and thereby avoid or delay expensive long-term institutional care.

Decision making to prevent and more effectively manage chronic disease in vulnerable populations requires consideration of factors other than small-area socioeconomic deprivation. Palmetto SADI may be most valuable as a policy and program planning tool when combined with other small-area assessment strategies measuring such factors as healthy food availability [60], health care accessibility (remoteness) [25], health professional workforce supply [25], adequacy of health care provider education programs [61], health care utilization, and health care quality. The integration of Palmetto SADI with diverse data elements like these, especially in the context of a geographic information system (GIS), could strengthen efforts to locate at-risk populations, identify gaps between health need and available health care and other community resources, target program initiatives, and encourage stakeholder collaboration to promote population health and reduce health disparities over time and space.

## Conclusions

As a predictor of chronic disease burden among South Carolina Medicaid recipients, Palmetto SADI outperformed all alternative small-area deprivation measures tested. Palmetto SADI can be used to identify areas in South Carolina at high risk for chronic disease burden among Medicaid recipients and other low-income Medicaid-eligible populations for targeted prevention, disease management, and care coordination activities.
